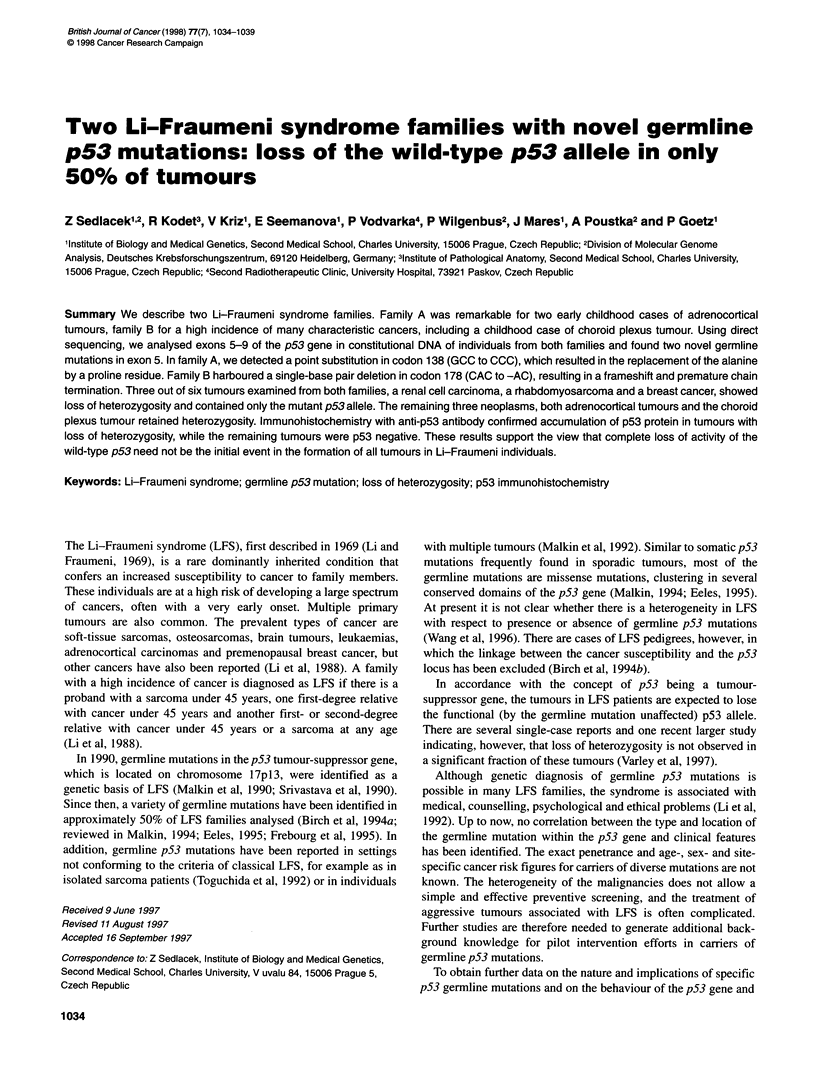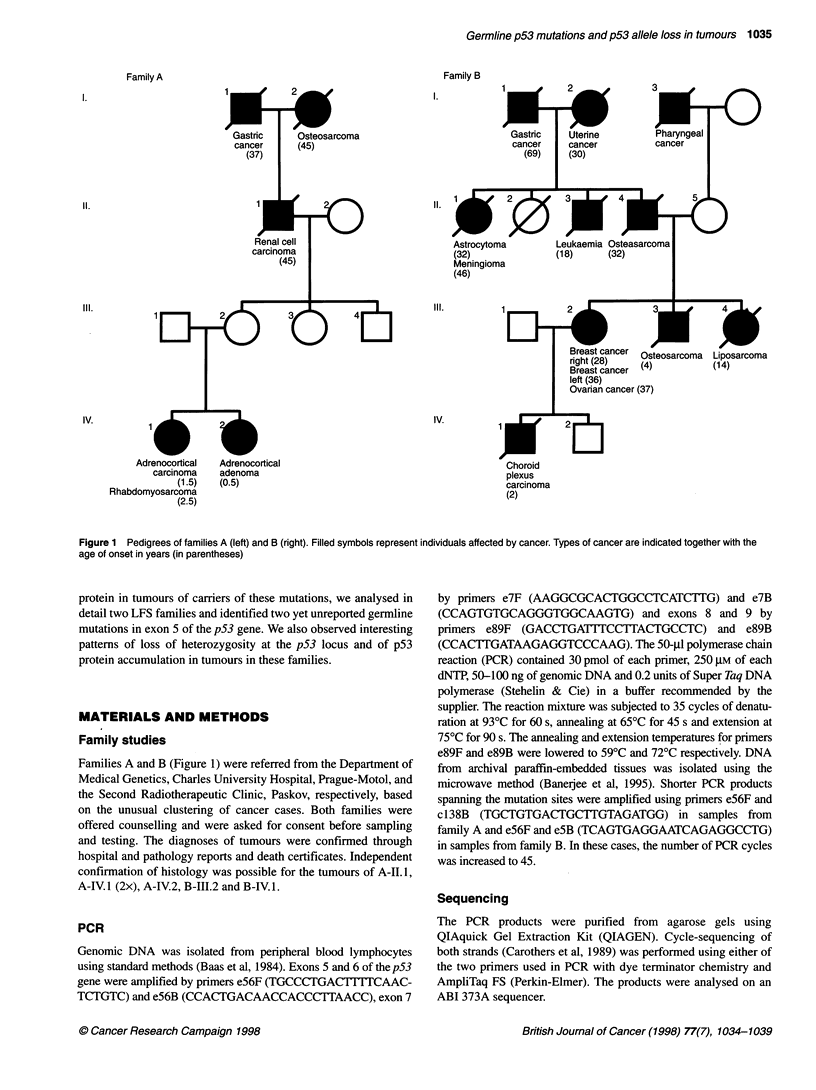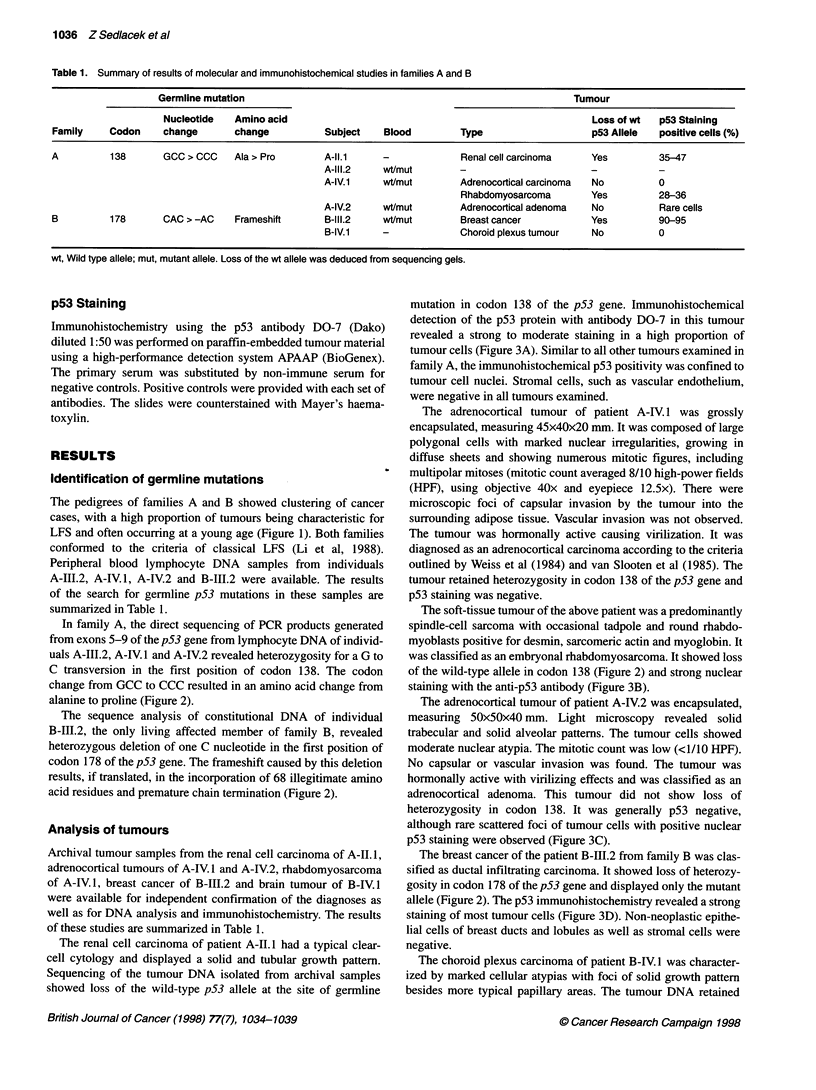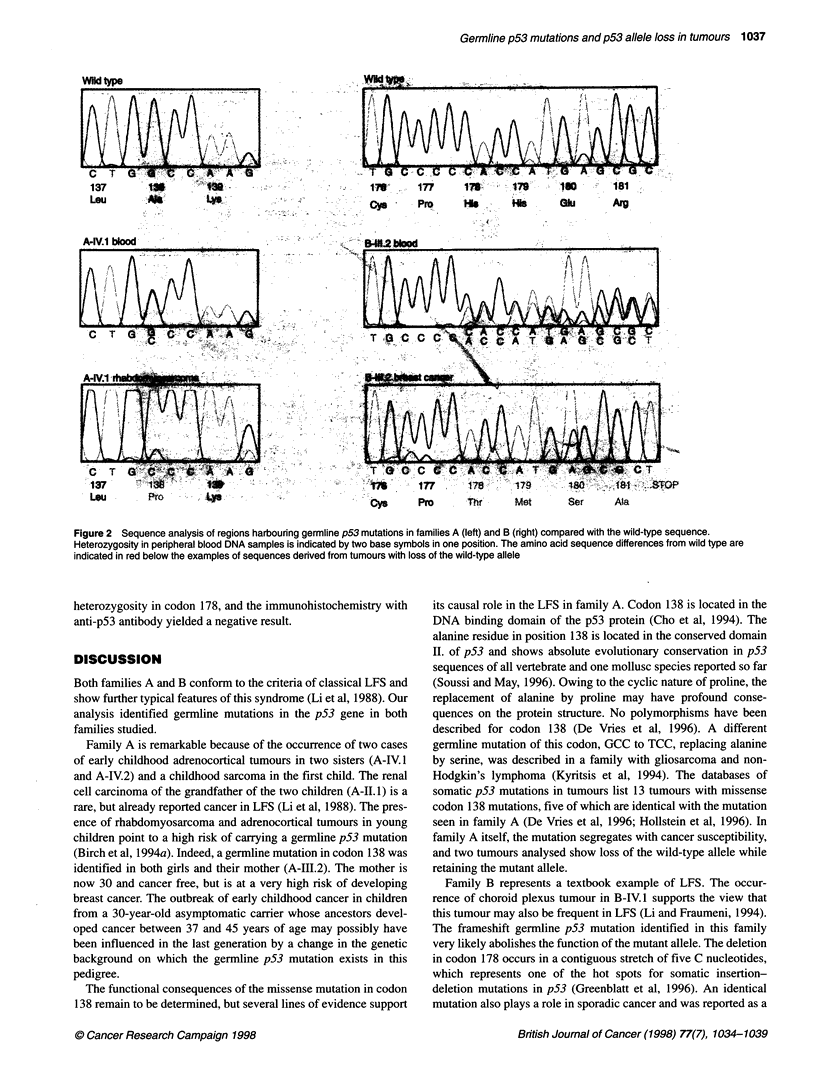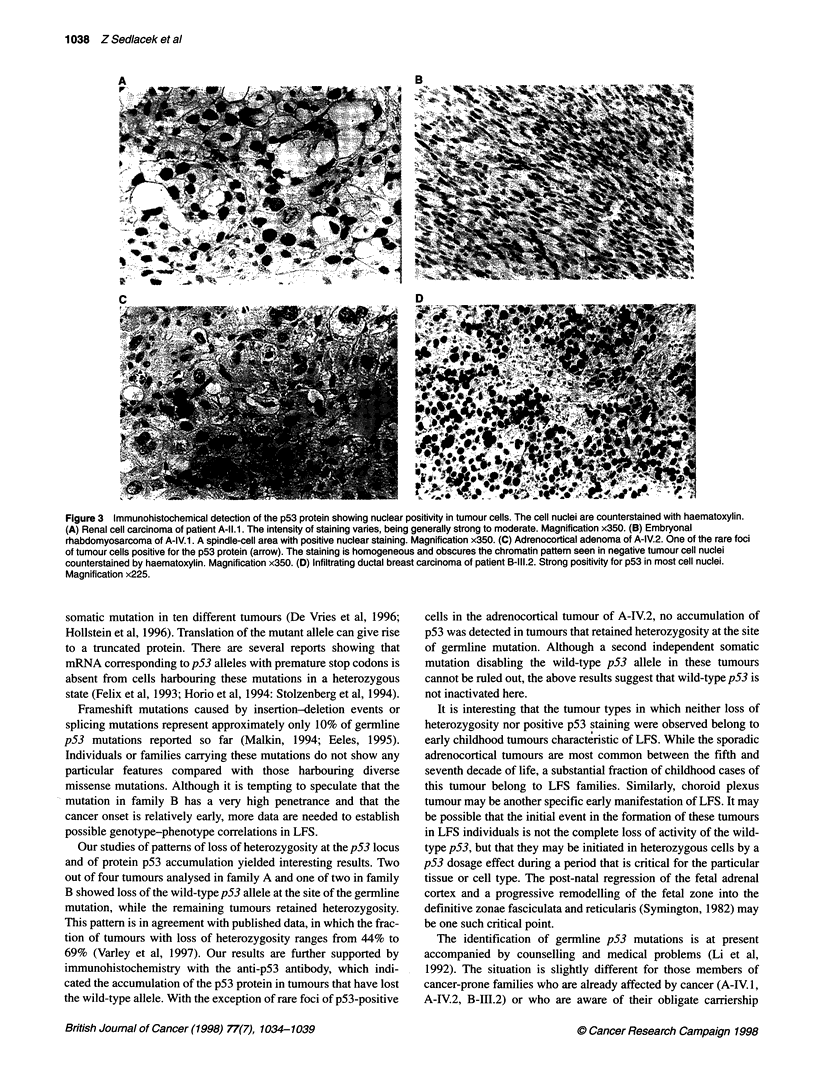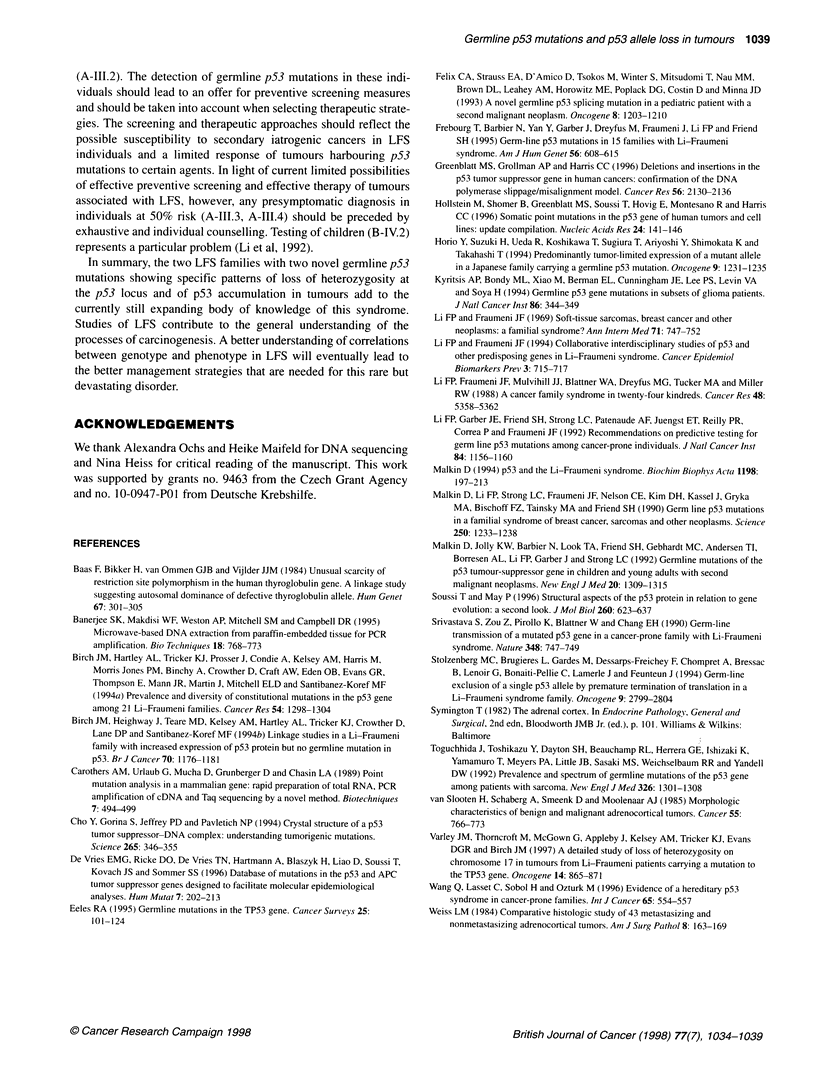# Two Li-Fraumeni syndrome families with novel germline p53 mutations: loss of the wild-type p53 allele in only 50% of tumours.

**DOI:** 10.1038/bjc.1998.172

**Published:** 1998-04

**Authors:** Z. Sedlacek, R. Kodet, V. Kriz, E. Seemanova, P. Vodvarka, P. Wilgenbus, J. Mares, A. Poustka, P. Goetz

**Affiliations:** Institute of Biology and Medical Genetics, Second Medical School, Charles University, Prague, Czech Republic.

## Abstract

**Images:**


British Journal of Cancer (1998) 77(7), 1034–1039
© 1998 Cancer Research Campaign

### Two Li–Fraumeni syndrome families with novel germline *p53* mutations: loss of the wild-type *p53* allele in only 50% of tumours

**Z Sedlacek[1,2], R Kodet[3], V Kriz[1], E Seemanova[1], P Vodvarka[4], P Wilgenbus[2], J Mares[1], A Poustka[2] and P Goetz[1]**

[1]Institute of Biology and Medical Genetics, Second Medical School, Charles University, 15006 Prague, Czech Republic; [2]Division of Molecular Genome Analysis, Deutsches Krebsforschungszentrum, 69120 Heidelberg, Germany; [3]Institute of Pathological Anatomy, Second Medical School, Charles University, 15006 Prague, Czech Republic; [4]Second Radiotherapeutic Clinic, University Hospital, 73921 Paskov, Czech Republic

**Summary** We describe two Li–Fraumeni syndrome families. Family A was remarkable for two early childhood cases of adrenocortical tumours, family B for a high incidence of many characteristic cancers, including a childhood case of choroid plexus tumour. Using direct sequencing, we analysed exons 5–9 of the *p53* gene in constitutional DNA of individuals from both families and found two novel germline mutations in exon 5. In family A, we detected a point substitution in codon 138 (GCC to CCC), which resulted in the replacement of the alanine by a proline residue. Family B harboured a single-base pair deletion in codon 178 (CAC to –AC), resulting in a frameshift and premature chain termination. Three out of six tumours examined from both families, a renal cell carcinoma, a rhabdomyosarcoma and a breast cancer, showed loss of heterozygosity and contained only the mutant *p53* allele. The remaining three neoplasms, both adrenocortical tumours and the choroid plexus tumour retained heterozygosity. Immunohistochemistry with anti-p53 antibody confirmed accumulation of p53 protein in tumours with loss of heterozygosity, while the remaining tumours were p53 negative. These results support the view that complete loss of activity of the wild-type *p53* need not be the initial event in the formation of all tumours in Li–Fraumeni individuals.

**Keywords:** Li–Fraumeni syndrome; germline *p53* mutation; loss of heterozygosity; p53 immunohistochemistry

The Li–Fraumeni syndrome (LFS), first described in 1969 (Li and Fraumeni, 1969), is a rare dominantly inherited condition that confers an increased susceptibility to cancer to family members. These individuals are at a high risk of developing a large spectrum of cancers, often with a very early onset. Multiple primary tumours are also common. The prevalent types of cancer are soft-tissue sarcomas, osteosarcomas, brain tumours, leukaemias, adrenocortical carcinomas and premenopausal breast cancer, but other cancers have also been reported (Li et al, 1988). A family with a high incidence of cancer is diagnosed as LFS if there is a proband with a sarcoma under 45 years, one first-degree relative with cancer under 45 years and another first- or second-degree relative with cancer under 45 years or a sarcoma at any age (Li et al, 1988).

In 1990, germline mutations in the *p53* tumour-suppressor gene, which is located on chromosome 17p13, were identified as a genetic basis of LFS (Malkin et al, 1990; Srivastava et al, 1990). Since then, a variety of germline mutations have been identified in approximately 50% of LFS families analysed (Birch et al, 1994*a*; reviewed in Malkin, 1994; Eeles, 1995; Frebourg et al, 1995). In addition, germline *p53* mutations have been reported in settings not conforming to the criteria of classical LFS, for example as in isolated sarcoma patients (Toguchida et al, 1992) or in individuals with multiple tumours (Malkin et al, 1992). Similar to somatic *p53* mutations frequently found in sporadic tumours, most of the germline mutations are missense mutations, clustering in several conserved domains of the *p53* gene (Malkin, 1994; Eeles, 1995). At present it is not clear whether there is a heterogeneity in LFS with respect to presence or absence of germline *p53* mutations (Wang et al, 1996). There are cases of LFS pedigrees, however, in which the linkage between the cancer susceptibility and the *p53* locus has been excluded (Birch et al, 1994*b*).

In accordance with the concept of *p53* being a tumour-suppressor gene, the tumours in LFS patients are expected to lose the functional (by the germline mutation unaffected) p53 allele. There are several single-case reports and one recent larger study indicating, however, that loss of heterozygosity is not observed in a significant fraction of these tumours (Varley et al, 1997).

Although genetic diagnosis of germline *p53* mutations is possible in many LFS families, the syndrome is associated with medical, counselling, psychological and ethical problems (Li et al, 1992). Up to now, no correlation between the type and location of the germline mutation within the *p53* gene and clinical features has been identified. The exact penetrance and age-, sex- and site-specific cancer risk figures for carriers of diverse mutations are not known. The heterogeneity of the malignancies does not allow a simple and effective preventive screening, and the treatment of aggressive tumours associated with LFS is often complicated. Further studies are therefore needed to generate additional background knowledge for pilot intervention efforts in carriers of germline *p53* mutations.

To obtain further data on the nature and implications of specific *p53* germline mutations and on the behaviour of the *p53* gene and

*Received 9 June 1997*
*Revised 11 August 1997*
*Accepted 16 September 1997*

*Correspondence to:* Z Sedlacek, Institute of Biology and Medical Genetics, Second Medical School, Charles University, V uvalu 84, 15006 Prague 5, Czech Republic

**Figure 1** Pedigrees of families A (left) and B (right). Filled symbols represent individuals affected by cancer. Types of cancer are indicated together with the age of onset in years (in parentheses)

protein in tumours of carriers of these mutations, we analysed in detail two LFS families and identified two yet unreported germline mutations in exon 5 of the *p53* gene. We also observed interesting patterns of loss of heterozygosity at the *p53* locus and of p53 protein accumulation in tumours in these families.

### MATERIALS AND METHODS

### Family studies

Families A and B (Figure 1) were referred from the Department of Medical Genetics, Charles University Hospital, Prague-Motol, and the Second Radiotherapeutic Clinic, Paskov, respectively, based on the unusual clustering of cancer cases. Both families were offered counselling and were asked for consent before sampling and testing. The diagnoses of tumours were confirmed through hospital and pathology reports and death certificates. Independent confirmation of histology was possible for the tumours of A-II.1, A-IV.1 (2×), A-IV.2, B-III.2 and B-IV.1.

### PCR

Genomic DNA was isolated from peripheral blood lymphocytes using standard methods (Baas et al, 1984). Exons 5 and 6 of the *p53* gene were amplified by primers e56F (TGCCCTGACTTTTCAAC-TCTGTC) and e56B (CCACTGACAACCACCCTTAACC), exon 7 by primers e7F (AAGGCGCACTGGCCTCATCTTG) and e7B (CCAGTGTGCAGGGTGGCAAGTG) and exons 8 and 9 by primers e89F (GACCTGATTTCCTTACTGCCTC) and e89B (CCACTTGATAAGAGGTCCCAAG). The 50-μl polymerase chain reaction (PCR) contained 30 pmol of each primer, 250 μM of each dNTP, 50–100 ng of genomic DNA and 0.2 units of Super *Taq* DNA polymerase (Stehelin & Cie) in a buffer recommended by the supplier. The reaction mixture was subjected to 35 cycles of denaturation at 93°C for 60 s, annealing at 65°C for 45 s and extension at 75°C for 90 s. The annealing and extension temperatures for primers e89F and e89B were lowered to 59°C and 72°C respectively. DNA from archival paraffin-embedded tissues was isolated using the microwave method (Banerjee et al, 1995). Shorter PCR products spanning the mutation sites were amplified using primers e56F and c138B (TGCTGTGACTGCTTGTAGATGG) in samples from family A and e56F and e5B (TCAGTGAGGAATCAGAGGCCTG) in samples from family B. In these cases, the number of PCR cycles was increased to 45.

### Sequencing

The PCR products were purified from agarose gels using QIAquick Gel Extraction Kit (QIAGEN). Cycle-sequencing of both strands (Carothers et al, 1989) was performed using either of the two primers used in PCR with dye terminator chemistry and AmpliTaq FS (Perkin-Elmer). The products were analysed on an ABI 373A sequencer.

© Cancer Research Campaign 1998

Table 1. Summary of results of molecular and immunohistochemical studies in families A and B

| | Germline mutation | | | | | Tumour | | |
|---|---|---|---|---|---|---|---|---|
| Family | Codon | Nucleotide change | Amino acid change | Subject | Blood | Type | Loss of wt p53 Allele | p53 Staining positive cells (%) |
| A | 138 | GCC > CCC | Ala > Pro | A-II.1 | – | Renal cell carcinoma | Yes | 35–47 |
| | | | | A-III.2 | wt/mut | – | – | – |
| | | | | A-IV.1 | wt/mut | Adrenocortical carcinoma | No | 0 |
| | | | | | | Rhabdomyosarcoma | Yes | 28–36 |
| | | | | A-IV.2 | wt/mut | Adrenocortical adenoma | No | Rare cells |
| B | 178 | CAC > –AC | Frameshift | B-III.2 | wt/mut | Breast cancer | Yes | 90–95 |
| | | | | B-IV.1 | – | Choroid plexus tumour | No | 0 |

wt, Wild type allele; mut, mutant allele. Loss of the wt allele was deduced from sequencing gels.

### p53 Staining

Immunohistochemistry using the p53 antibody DO-7 (Dako) diluted 1:50 was performed on paraffin-embedded tumour material using a high-performance detection system APAAP (BioGenex). The primary serum was substituted by non-immune serum for negative controls. Positive controls were provided with each set of antibodies. The slides were counterstained with Mayer's haematoxylin.

### RESULTS

### Identification of germline mutations

The pedigrees of families A and B showed clustering of cancer cases, with a high proportion of tumours being characteristic for LFS and often occurring at a young age (Figure 1). Both families conformed to the criteria of classical LFS (Li et al, 1988). Peripheral blood lymphocyte DNA samples from individuals A-III.2, A-IV.1, A-IV.2 and B-III.2 were available. The results of the search for germline *p53* mutations in these samples are summarized in Table 1.

In family A, the direct sequencing of PCR products generated from exons 5–9 of the *p53* gene from lymphocyte DNA of individuals A-III.2, A-IV.1 and A-IV.2 revealed heterozygosity for a G to C transversion in the first position of codon 138. The codon change from GCC to CCC resulted in an amino acid change from alanine to proline (Figure 2).

The sequence analysis of constitutional DNA of individual B-III.2, the only living affected member of family B, revealed heterozygous deletion of one C nucleotide in the first position of codon 178 of the *p53* gene. The frameshift caused by this deletion results, if translated, in the incorporation of 68 illegitimate amino acid residues and premature chain termination (Figure 2).

### Analysis of tumours

Archival tumour samples from the renal cell carcinoma of A-II.1, adrenocortical tumours of A-IV.1 and A-IV.2, rhabdomyosarcoma of A-IV.1, breast cancer of B-III.2 and brain tumour of B-IV.1 were available for independent confirmation of the diagnoses as well as for DNA analysis and immunohistochemistry. The results of these studies are summarized in Table 1.

The renal cell carcinoma of patient A-II.1 had a typical clear-cell cytology and displayed a solid and tubular growth pattern. Sequencing of the tumour DNA isolated from archival samples showed loss of the wild-type *p53* allele at the site of germline mutation in codon 138 of the *p53* gene. Immunohistochemical detection of the p53 protein with antibody DO-7 in this tumour revealed a strong to moderate staining in a high proportion of tumour cells (Figure 3A). Similar to all other tumours examined in family A, the immunohistochemical p53 positivity was confined to tumour cell nuclei. Stromal cells, such as vascular endothelium, were negative in all tumours examined.

The adrenocortical tumour of patient A-IV.1 was grossly encapsulated, measuring 45×40×20 mm. It was composed of large polygonal cells with marked nuclear irregularities, growing in diffuse sheets and showing numerous mitotic figures, including multipolar mitoses (mitotic count averaged 8/10 high-power fields (HPF), using objective 40× and eyepiece 12.5×). There were microscopic foci of capsular invasion by the tumour into the surrounding adipose tissue. Vascular invasion was not observed. The tumour was hormonally active causing virilization. It was diagnosed as an adrenocortical carcinoma according to the criteria outlined by Weiss et al (1984) and van Slooten et al (1985). The tumour retained heterozygosity in codon 138 of the *p53* gene and p53 staining was negative.

The soft-tissue tumour of the above patient was a predominantly spindle-cell sarcoma with occasional tadpole and round rhabdomyoblasts positive for desmin, sarcomeric actin and myoglobin. It was classified as an embryonal rhabdomyosarcoma. It showed loss of the wild-type allele in codon 138 (Figure 2) and strong nuclear staining with the anti-p53 antibody (Figure 3B).

The adrenocortical tumour of patient A-IV.2 was encapsulated, measuring 50×50×40 mm. Light microscopy revealed solid trabecular and solid alveolar patterns. The tumour cells showed moderate nuclear atypia. The mitotic count was low (<1/10 HPF). No capsular or vascular invasion was found. The tumour was hormonally active with virilizing effects and was classified as an adrenocortical adenoma. This tumour did not show loss of heterozygosity in codon 138. It was generally p53 negative, although rare scattered foci of tumour cells with positive nuclear p53 staining were observed (Figure 3C).

The breast cancer of the patient B-III.2 from family B was classified as ductal infiltrating carcinoma. It showed loss of heterozygosity in codon 178 of the *p53* gene and displayed only the mutant allele (Figure 2). The p53 immunohistochemistry revealed a strong staining of most tumour cells (Figure 3D). Non-neoplastic epithelial cells of breast ducts and lobules as well as stromal cells were negative.

The choroid plexus carcinoma of patient B-IV.1 was characterized by marked cellular atypias with foci of solid growth pattern besides more typical papillary areas. The tumour DNA retained

© Cancer Research Campaign 1998

**Figure 2** Sequence analysis of regions harbouring germline *p53* mutations in families A (left) and B (right) compared with the wild-type sequence. Heterozygosity in peripheral blood DNA samples is indicated by two base symbols in one position. The amino acid sequence differences from wild type are indicated in red below the examples of sequences derived from tumours with loss of the wild-type allele heterozygosity in codon 178, and the immunohistochemistry with anti-p53 antibody yielded a negative result.

### DISCUSSION

Both families A and B conform to the criteria of classical LFS and show further typical features of this syndrome (Li et al, 1988). Our analysis identified germline mutations in the *p53* gene in both families studied.

Family A is remarkable because of the occurrence of two cases of early childhood adrenocortical tumours in two sisters (A-IV.1 and A-IV.2) and a childhood sarcoma in the first child. The renal cell carcinoma of the grandfather of the two children (A-II.1) is a rare, but already reported cancer in LFS (Li et al, 1988). The presence of rhabdomyosarcoma and adrenocortical tumours in young children point to a high risk of carrying a germline *p53* mutation (Birch et al, 1994*a*). Indeed, a germline mutation in codon 138 was identified in both girls and their mother (A-III.2). The mother is now 30 and cancer free, but is at a very high risk of developing breast cancer. The outbreak of early childhood cancer in children from a 30-year-old asymptomatic carrier whose ancestors developed cancer between 37 and 45 years of age may possibly have been influenced in the last generation by a change in the genetic background on which the germline *p53* mutation exists in this pedigree.

The functional consequences of the missense mutation in codon 138 remain to be determined, but several lines of evidence support its causal role in the LFS in family A. Codon 138 is located in the DNA binding domain of the p53 protein (Cho et al, 1994). The alanine residue in position 138 is located in the conserved domain II. of *p53* and shows absolute evolutionary conservation in *p53* sequences of all vertebrate and one mollusc species reported so far (Soussi and May, 1996). Owing to the cyclic nature of proline, the replacement of alanine by proline may have profound consequences on the protein structure. No polymorphisms have been described for codon 138 (De Vries et al, 1996). A different germline mutation of this codon, GCC to TCC, replacing alanine by serine, was described in a family with gliosarcoma and non-Hodgkin's lymphoma (Kyritsis et al, 1994). The databases of somatic *p53* mutations in tumours list 13 tumours with missense codon 138 mutations, five of which are identical with the mutation seen in family A (De Vries et al, 1996; Hollstein et al, 1996). In family A itself, the mutation segregates with cancer susceptibility, and two tumours analysed show loss of the wild-type allele while retaining the mutant allele.

Family B represents a textbook example of LFS. The occurrence of choroid plexus tumour in B-IV.1 supports the view that this tumour may also be frequent in LFS (Li and Fraumeni, 1994). The frameshift germline *p53* mutation identified in this family very likely abolishes the function of the mutant allele. The deletion in codon 178 occurs in a contiguous stretch of five C nucleotides, which represents one of the hot spots for somatic insertion–deletion mutations in *p53* (Greenblatt et al, 1996). An identical mutation also plays a role in sporadic cancer and was reported as a

© Cancer Research Campaign 1998

**Figure 3** Immunohistochemical detection of the p53 protein showing nuclear positivity in tumour cells. The cell nuclei are counterstained with haematoxylin. (**A**) Renal cell carcinoma of patient A-II.1. The intensity of staining varies, being generally strong to moderate. Magnification ×350. (**B**) Embryonal rhabdomyosarcoma of A-IV.1. A spindle-cell area with positive nuclear staining. Magnification ×350. (**C**) Adrenocortical adenoma of A-IV.2. One of the rare foci of tumour cells positive for the p53 protein (arrow). The staining is homogeneous and obscures the chromatin pattern seen in negative tumour cell nuclei counterstained by haematoxylin. Magnification ×350. (**D**) Infiltrating ductal breast carcinoma of patient B-III.2. Strong positivity for p53 in most cell nuclei. Magnification ×225.

somatic mutation in ten different tumours (De Vries et al, 1996; Hollstein et al, 1996). Translation of the mutant allele can give rise to a truncated protein. There are several reports showing that mRNA corresponding to *p53* alleles with premature stop codons is absent from cells harbouring these mutations in a heterozygous state (Felix et al, 1993; Horio et al, 1994: Stolzenberg et al, 1994).

Frameshift mutations caused by insertion–deletion events or splicing mutations represent approximately only 10% of germline *p53* mutations reported so far (Malkin, 1994; Eeles, 1995). Individuals or families carrying these mutations do not show any particular features compared with those harbouring diverse missense mutations. Although it is tempting to speculate that the mutation in family B has a very high penetrance and that the cancer onset is relatively early, more data are needed to establish possible genotype–phenotype correlations in LFS.

Our studies of patterns of loss of heterozygosity at the *p53* locus and of protein p53 accumulation yielded interesting results. Two out of four tumours analysed in family A and one of two in family B showed loss of the wild-type *p53* allele at the site of the germline mutation, while the remaining tumours retained heterozygosity. This pattern is in agreement with published data, in which the fraction of tumours with loss of heterozygosity ranges from 44% to 69% (Varley et al, 1997). Our results are further supported by immunohistochemistry with the anti-p53 antibody, which indicated the accumulation of the p53 protein in tumours that have lost the wild-type allele. With the exception of rare foci of p53-positive cells in the adrenocortical tumour of A-IV.2, no accumulation of p53 was detected in tumours that retained heterozygosity at the site of germline mutation. Although a second independent somatic mutation disabling the wild-type *p53* allele in these tumours cannot be ruled out, the above results suggest that wild-type *p53* is not inactivated here.

It is interesting that the tumour types in which neither loss of heterozygosity nor positive p53 staining were observed belong to early childhood tumours characteristic of LFS. While the sporadic adrenocortical tumours are most common between the fifth and seventh decade of life, a substantial fraction of childhood cases of this tumour belong to LFS families. Similarly, choroid plexus tumour may be another specific early manifestation of LFS. It may be possible that the initial event in the formation of these tumours in LFS individuals is not the complete loss of activity of the wild-type *p53*, but that they may be initiated in heterozygous cells by a *p53* dosage effect during a period that is critical for the particular tissue or cell type. The post-natal regression of the fetal adrenal cortex and a progressive remodelling of the fetal zone into the definitive zonae fasciculata and reticularis (Symington, 1982) may be one such critical point.

The identification of germline *p53* mutations is at present accompanied by counselling and medical problems (Li et al, 1992). The situation is slightly different for those members of cancer-prone families who are already affected by cancer (A-IV.1, A-IV.2, B-III.2) or who are aware of their obligate carriership

© Cancer Research Campaign 1998

(A-III.2). The detection of germline *p53* mutations in these individuals should lead to an offer for preventive screening measures and should be taken into account when selecting therapeutic strategies. The screening and therapeutic approaches should reflect the possible susceptibility to secondary iatrogenic cancers in LFS individuals and a limited response of tumours harbouring *p53* mutations to certain agents. In light of current limited possibilities of effective preventive screening and effective therapy of tumours associated with LFS, however, any presymptomatic diagnosis in individuals at 50% risk (A-III.3, A-III.4) should be preceded by exhaustive and individual counselling. Testing of children (B-IV.2) represents a particular problem (Li et al, 1992).

In summary, the two LFS families with two novel germline *p53* mutations showing specific patterns of loss of heterozygosity at the *p53* locus and of p53 accumulation in tumours add to the currently still expanding body of knowledge of this syndrome. Studies of LFS contribute to the general understanding of the processes of carcinogenesis. A better understanding of correlations between genotype and phenotype in LFS will eventually lead to the better management strategies that are needed for this rare but devastating disorder.

### ACKNOWLEDGEMENTS

We thank Alexandra Ochs and Heike Maifeld for DNA sequencing and Nina Heiss for critical reading of the manuscript. This work was supported by grants no. 9463 from the Czech Grant Agency and no. 10-0947-P01 from Deutsche Krebshilfe.

### REFERENCES

Baas F, Bikker H, van Ommen GJB and Vijlder JJM (1984) Unusual scarcity of restriction site polymorphism in the human thyroglobulin gene. A linkage study suggesting autosomal dominance of defective thyroglobulin allele. *Hum Genet* **67**: 301–305

Banerjee SK, Makdisi WF, Weston AP, Mitchell SM and Campbell DR (1995) Microwave-based DNA extraction from paraffin-embedded tissue for PCR amplification. *Bio Techniques* **18**: 768–773

Birch JM, Hartley AL, Tricker KJ, Prosser J, Condie A, Kelsey AM, Harris M, Morris Jones PM, Binchy A, Crowther D, Craft AW, Eden OB, Evans GR, Thompson E, Mann JR, Martin J, Mitchell ELD and Santibanez-Koref MF (1994*a*) Prevalence and diversity of constitutional mutations in the p53 gene among 21 Li–Fraumeni families. *Cancer Res* **54**: 1298–1304

Birch JM, Heighway J, Teare MD, Kelsey AM, Hartley AL, Tricker KJ, Crowther D, Lane DP and Santibanez-Koref MF (1994*b*) Linkage studies in a Li–Fraumeni family with increased expression of p53 protein but no germline mutation in p53. *Br J Cancer* **70**: 1176–1181

Carothers AM, Urlaub G, Mucha D, Grunberger D and Chasin LA (1989) Point mutation analysis in a mammalian gene: rapid preparation of total RNA, PCR amplification of cDNA and Taq sequencing by a novel method. *Biotechniques* **7**: 494–499

Cho Y, Gorina S, Jeffrey PD and Pavletich NP (1994) Crystal structure of a p53 tumor suppressor–DNA complex: understanding tumorigenic mutations. *Science* **265**: 346–355

De Vries EMG, Ricke DO, De Vries TN, Hartmann A, Blaszyk H, Liao D, Soussi T, Kovach JS and Sommer SS (1996) Database of mutations in the p53 and APC tumor suppressor genes designed to facilitate molecular epidemiological analyses. *Hum Mutat* **7**: 202–213

Eeles RA (1995) Germline mutations in the TP53 gene. *Cancer Surveys* **25**: 101–124

Felix CA, Strauss EA, D'Amico D, Tsokos M, Winter S, Mitsudomi T, Nau MM, Brown DL, Leahey AM, Horowitz ME, Poplack DG, Costin D and Minna JD (1993) A novel germline p53 splicing mutation in a pediatric patient with a second malignant neoplasm. *Oncogene* **8**: 1203–1210

Frebourg T, Barbier N, Yan Y, Garber J, Dreyfus M, Fraumeni J, Li FP and Friend SH (1995) Germ-line p53 mutations in 15 families with Li–Fraumeni syndrome. *Am J Hum Genet* **56**: 608–615

Greenblatt MS, Grollman AP and Harris CC (1996) Deletions and insertions in the p53 tumor suppressor gene in human cancers: confirmation of the DNA polymerase slippage/misalignment model. *Cancer Res* **56**: 2130–2136

Hollstein M, Shomer B, Greenblatt MS, Soussi T, Hovig E, Montesano R and Harris CC (1996) Somatic point mutations in the p53 gene of human tumors and cell lines: update compilation. *Nucleic Acids Res* **24**: 141–146

Horio Y, Suzuki H, Ueda R, Koshikawa T, Sugiura T, Ariyoshi Y, Shimokata K and Takahashi T (1994) Predominantly tumor-limited expression of a mutant allele in a Japanese family carrying a germline p53 mutation. *Oncogene* **9**: 1231–1235

Kyritsis AP, Bondy ML, Xiao M, Berman EL, Cunningham JE, Lee PS, Levin VA and Soya H (1994) Germline p53 gene mutations in subsets of glioma patients. *J Natl Cancer Inst* **86**: 344–349

Li FP and Fraumeni JF (1969) Soft-tissue sarcomas, breast cancer and other neoplasms: a familial syndrome? *Ann Intern Med* **71**: 747–752

Li FP and Fraumeni JF (1994) Collaborative interdisciplinary studies of p53 and other predisposing genes in Li–Fraumeni syndrome. *Cancer Epidemiol Biomarkers Prev* **3**: 715–717

Li FP, Fraumeni JF, Mulvihill JJ, Blattner WA, Dreyfus MG, Tucker MA and Miller RW (1988) A cancer family syndrome in twenty-four kindreds. *Cancer Res* **48**: 5358–5362

Li FP, Garber JE, Friend SH, Strong LC, Patenaude AF, Juengst ET, Reilly PR, Correa P and Fraumeni JF (1992) Recommendations on predictive testing for germ line p53 mutations among cancer-prone individuals. *J Natl Cancer Inst* **84**: 1156–1160

Malkin D (1994) p53 and the Li–Fraumeni syndrome. *Biochim Biophys Acta* **1198**: 197–213

Malkin D, Li FP, Strong LC, Fraumeni JF, Nelson CE, Kim DH, Kassel J, Gryka MA, Bischoff FZ, Tainsky MA and Friend SH (1990) Germ line p53 mutations in a familial syndrome of breast cancer, sarcomas and other neoplasms. *Science* **250**: 1233–1238

Malkin D, Jolly KW, Barbier N, Look TA, Friend SH, Gebhardt MC, Andersen TI, Borresen AL, Li FP, Garber J and Strong LC (1992) Germline mutations of the p53 tumour-suppressor gene in children and young adults with second malignant neoplasms. *New Engl J Med* **20**: 1309–1315

Soussi T and May P (1996) Structural aspects of the p53 protein in relation to gene evolution: a second look. *J Mol Biol* **260**: 623–637

Srivastava S, Zou Z, Pirollo K, Blattner W and Chang EH (1990) Germ-line transmission of a mutated p53 gene in a cancer-prone family with Li-Fraumeni syndrome. *Nature* **348**: 747–749

Stolzenberg MC, Brugieres L, Gardes M, Dessarps-Freichey F, Chompret A, Bressac B, Lenoir G, Bonaiti-Pellie C, Lamerle J and Feunteun J (1994) Germ-line exclusion of a single p53 allele by premature termination of translation in a Li–Fraumeni syndrome family. *Oncogene* **9**: 2799–2804

Symington T (1982) The adrenal cortex. In *Endocrine Pathology, General and Surgical*, 2nd edn, Bloodworth JMB Jr. (ed.), p. 101. Williams & Wilkins: Baltimore

Toguchhida J, Toshikazu Y, Dayton SH, Beauchamp RL, Herrera GE, Ishizaki K, Yamamuro T, Meyers PA, Little JB, Sasaki MS, Weichselbaum RR and Yandell DW (1992) Prevalence and spectrum of germline mutations of the p53 gene among patients with sarcoma. *New Engl J Med* **326**: 1301–1308

van Slooten H, Schaberg A, Smeenk D and Moolenaar AJ (1985) Morphologic characteristics of benign and malignant adrenocortical tumors. *Cancer* **55**: 766–773

Varley JM, Thorncroft M, McGown G, Appleby J, Kelsey AM, Tricker KJ, Evans DGR and Birch JM (1997) A detailed study of loss of heterozygosity on chromosome 17 in tumours from Li–Fraumeni patients carrying a mutation to the TP53 gene. *Oncogene* **14**: 865–871

Wang Q, Lasset C, Sobol H and Ozturk M (1996) Evidence of a hereditary p53 syndrome in cancer-prone families. *Int J Cancer* **65**: 554–557

Weiss LM (1984) Comparative histologic study of 43 metastasizing and nonmetastasizing adrenocortical tumors. *Am J Surg Pathol* **8**: 163–169

© Cancer Research Campaign 1998